# PD12 Building A Sustainable Future: Health Demands And Technological Horizons In Immunobiologicals

**DOI:** 10.1017/S0266462325101499

**Published:** 2025-12-29

**Authors:** Thaís Catão, Carla Gottgtroy, Amanda Marques, Julianna Varela, Adriana Danowski, Jenifer de Paula, Eliane dos Santos, Elvira Lago, Beatriz Fialho, Maria de Lourdes Maia

## Abstract

**Introduction:**

Health priority setting is a complex process, especially in governmental decision-making, for improving access and addressing unmet needs. To review its technological roadmap, Bio-Manguinhos (BM)/Fiocruz has improved its strategy. The objective of this approach was to incorporate research institutions and medical and scientific experts into the technology analysis processes of a public pharmaceutical organization.

**Methods:**

The first stage of this roadmap review consisted of workshops in the areas of pneumology, infectious diseases, oncology, hematology, gastroenterology, dermatology, rheumatology, cardiology, and neurology. At each workshop, a scientific leader, researchers, and specialists discussed the main diseases and their short-, medium- and long-term health demands. These observations were translated into products and lines of health research by BM’s technical team. Epidemiological data were collected, and in-depth analysis was conducted that considered the type of technology, development status, and correlation with existing public policies. The products and lines of research were categorized, and a database was built for further examination in subsequent stages.

**Results:**

The 83 invited specialists highlighted 1,045 demands, of which 56 percent were long term, 39 percent short term, and 5 percent medium term. Market available products comprised 36 percent, while 51 percent were innovative solutions. Biotechnological demands represented 65 percent of the total, with 43 percent being biopharmaceuticals. Of those, 35 percent pertained to in vitro diagnostics, nine percent to vaccines, and 13 percent to other technologies. Monoclonal antibodies stood out at 24 percent. Cellular therapies made up six percent, gene therapies two percent, chimeric antigen receptor T-cell therapies two percent, and antibody-drug conjugates one percent. Rheumatology had the highest number of biotechnological demands and accounted for 24 percent.

**Conclusions:**

The importance of recognizing genetic and economic patterns in the diseases that most affect the population, with a focus on personalized medicine, was consistently emphasized. Health demand assessment is a form of social participation in the decision-making process. Combined with other data sources, it allows for integrated and synergistic mapping to analyze existing biotechnological products and innovative solutions.

